# Rotenone Directly Induces BV2 Cell Activation via the p38 MAPK Pathway

**DOI:** 10.1371/journal.pone.0072046

**Published:** 2013-08-20

**Authors:** Feng Gao, Dong Chen, Qingsong Hu, Guanghui Wang

**Affiliations:** 1 Laboratory of Molecular Neuropathology, Key Laboratory of Brain Function and Diseases and School of Life Sciences, University of Science & Technology of China, Chinese Academy of Sciences, Hefei, China; 2 Laboratory of Molecular Neuropathology, Department of Pharmacology, Soochow University College of Pharmaceutical Sciences, Suzhou, China; Baylor College of Medicine, Jiao Tong University School of Medicine, United States of America

## Abstract

Parkinson’s disease (PD) is the second most common neurodegenerative disease. Although its pathogenesis is still unclear, increasing evidence suggests that mitochondrial dysfunction induced by environmental toxins, such as mitochondrial complex I inhibitors, plays a significant role in the disease process. The microglia in PD brains are highly activated, and inflammation is also an essential element in PD pathogenesis. However, the means by which these toxins activate microglia is still unclear. In the present study, we found that rotenone, a mitochondrial complex I inhibitor, could directly activate microglia via the nuclear factor kappa B (NF-κB) signaling pathway, thereby inducing significantly increased expression of inflammatory cytokines. We further observed that rotenone induced caspase-1 activation and mature IL-1β release, both of which are strictly dependent on p38 mitogen-activated protein kinase (MAPK). The activation of p38 is associated with the presence of reactive oxygen species (ROS) produced by rotenone. Removal of these ROS abrogated the activation of the microglia. Therefore, our data suggest that the environmental toxin rotenone can directly activate microglia through the p38 MAPK pathway.

## Introduction

Parkinson’ disease (PD) is one of the most common neurodegenerative diseases [1]. Its pathological hallmarks include the preferential loss of dopaminergic (DA) neurons in the substantia nigra pars compacta (SNpc) [2]. Although some gene mutations have been implicated in the pathogenesis of PD [3], [4], most PD cases are sporadic. In these patients, mitochondrial dysfunction induced by environmental toxins is considered a major etiology of PD [5]. The toxic effects of environmental toxins on DA neurons result from the inhibition of complex I of the mitochondrial electron transport chain, increased production of free radicals and excessive oxidative stress [6]. In experimental animals, administration of mitochondrial toxins, such as 1-methyl-4-phenyl-1,2,3,4-tetrahydropyridine (MPTP) or rotenone, can induce a PD-like syndrome characterized by DA neuronal degeneration [4]. Thus, these studies highlight the role of mitochondrial dysfunction in the pathogenesis of PD.

Interestingly, the presence of activated microglia has been observed, together with DA neuronal degeneration, in the SNpc of PD patients [7], [8], [9], [10]. Activated microglia and elevated expression of inflammatory factors, such as tumor necrosis factor α (TNFα) or interleukin (IL)-1β, have been observed in PD patients [11]. Postmortem studies of MPTP-intoxicated patients have also revealed sustained neuroinflammation in the SNpc [12], [13]. In addition, in an animal model of PD induced by MPTP or rotenone, activated microglia were also observed [14], [15].

Many studies have suggested that neuroinflammation plays an important role in the pathogenesis of PD [16], [17]. An epidemiologic study showed a reduced risk of developing the disease in individuals taking non-steroidal anti-inflammatory drugs [18]. Direct evidence demonstrating the association between neuroinflammation and DA neuronal injury came from a study in which lipopolysaccharide (LPS), an immunogen that activates the production of various neuroinflammatory factors in glial cells in the CNS, induced DA neuronal loss in the SNpc [19]. In addition, the combined administration of LPS and MPTP was also able to induce acute parkinsonian syndrome in the animals [17], [20]. However, when the inflammatory pathway was blocked, this treatment was able to rescue the DA neuronal loss in MPTP-treated animals [20]. Therefore, neuroinflammation plays a role in the pathogenesis of PD.

Although it is not clear what might drive inflammation in PD brains, recent studies suggest that components of the extracellular milieu that may be generated after neuronal injury can act as stimuli that activate astrocytes and microglia [8], [14], [20]. However, some studies have shown that microglia are activated before neuronal death, suggesting that the microglial activation may be independent of the release of toxic cytosolic compounds by the surrounding neurons [21]. Furthermore, our recent study shows that Omi, a PD-associated gene product, can regulate the MAPK (mitogen-activated protein kinase) signaling pathway to influence nuclear factor kappa B (NF-κB) activity. A defect in Omi protease activity actives NF-κB to produce inflammatory factors in microglia [22], further suggesting that the microglia may be activated independently of neuronal injury. Rotenone is an environmental toxin that inhibits mitochondrial complex I, thereby damaging DA neurons; however, it is unclear whether rotenone induces microglial activation.

In the present study, we found that rotenone, a mitochondria complex I inhibitor, significantly activated the NF-κB signaling pathway in the BV2 microglial cell line. The activated NF-κB signaling pathway induced significant production of inflammatory factors and activated inflammasomes, which are dependent on the MAPK p38. Rotenone damaged the mitochondria to produce reactive oxygen species (ROS), which induce p38 activation. Moreover, scavenging the ROS effectively blocked the microglial activation.

## Materials and Methods

### Cell Culture and Treatment

BV2 cells (a mouse microglial cell line was a kind gift from Dr. Jianqing Ding at Shanghai Jiao Tong University, China) [23] were cultured in Dulbecco’s modified Eagle’s medium (DMEM) (Invitrogen) containing 10% fetal bovine serum (Invitrogen) with 100 mg/ml penicillin and 100 mg/ml streptomycin (Invitrogen). BV2 cells were treated with LPS (Sigma-Aldrich) at a concentration of 1 µg/ml or rotenone (Sigma-Aldrich) at concentrations of 0.1, 0.25, 0.5, and 1 µM. Cells were also treated with N-acetylcysteine (NAC) (Sigma-Aldrich) at a concentration of 5 mM, either alone or together with rotenone or LPS. The Green FLICA™ Caspase-1 Assay Kit (Immunochemistry) was used to detect caspase-1 activation according to the manufacturer’s instructions. To detect ROS, DCFH-DA (Invitrogen) was added to the cell culture medium for 30 minutes.

### Subcellular Fractionation Assay for Nuclear Extraction

BV2 cells were lysed in fractionation buffer containing 320 mM sucrose, 3 mM CaCl_2,_ 2 mM MgAc, 0.1 mM EDTA, 1 mM DTT, 0.5 mM PMSF and 0.5% NP-40 for 20 min on ice. After centrifugation at 600 g for 15 min at 4°C, the supernatant was collected as the cytoplasmic fraction. The pellet was washed once with fractionation buffer without NP-40 and lysed in nuclear lysis buffer containing 20 mM HEPES at pH 7.9, 25% glycerol, 1.5 mM MgCl_2_, 280 mM KCl, 0.2 mM EDTA, 1 mM DTT, 0.5 mM PMSF and 0.3% NP-40.

### Immunoblot Analysis and Antibodies

The BV2 cells were lysed in RIPA buffer in the presence of a protease inhibitor cocktail (Roche). Approximately 20 µg of cell lysate was separated using SDS-PAGE and transferred onto a PVDF membrane (Millipore). Immunoblot analysis was performed with the following primary antibodies: polyclonal anti-iNOS (ABCAM); polyclonal anti-p65 (Epitomics); polyclonal anti-IL-1β (R&D); polyclonal anti-IKK, anti-pIKK(176/180), and anti-pp38 antibodies from Cell Signaling Technology; monoclonal anti-GAPDH antibody (Milipore); and monoclonal anti-p38 and anti-pERK1/2 antibody, and polyclonal anti-caspase-1, anti-ERK1/2 antibodies from Santa Cruz Biotechnology. The secondary antibodies, sheep anti-mouse or anti-rabbit IgG-HRP, were purchased from GE Health.

### ELISA Assay for TNF-α

The levels of TNF-α in 100 µl of cultured media of the treated BV2 cells were measured using ELISA kits (R&D) according to the manufacturer’s instructions.

### Immunocytofluorescence

The treated BV2 cells were cultured in chamber-slides and fixed with 4% paraformaldehyde in PBS (pH 7.4) for 5 minutes. The cells were then washed with PBS 3 times, and 0.25% Triton-X100 was added for 5 minutes. The cells were blocked with 3% FBS in PBST. Immunocytochemical staining was performed using polyclonal anti-p65 antibodies. After incubation with the primary antibody, the slides were incubated with Rho-conjugated donkey anti-mouse or anti-rabbit secondary antibodies (Jackson Immuno Research) and DAPI (Invitrogen).

## Results

### Rotenone Induced Degradation of IκB and Nuclear Accumulation of NF-κB p65 Subunit in BV2 Cells

**Figure 1:**
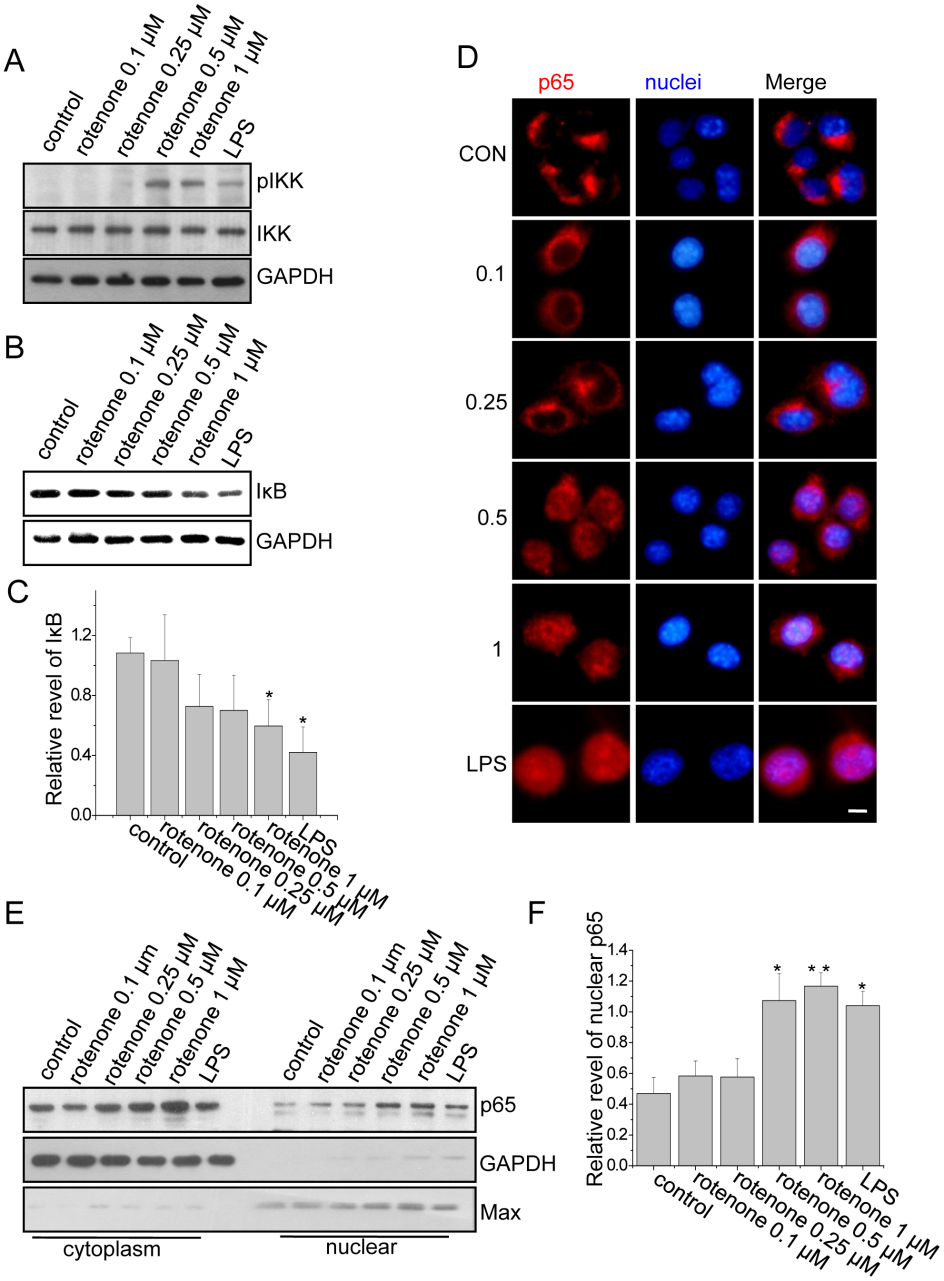
Rotenone directly activated the NF-κB signaling pathway in BV2 cells. (A) Various doses of rotenone or LPS were administered to BV2 cells as indicated for 6 hours or 1 hour, respectively. The cell lysates were immunoblotted with anti-pIKK or anti-IKK antibodies. GAPDH served as the loading control. (B) BV2 cells were treated as described in (A). The cell lysates were immunoblotted with anti-IκB antibodies. (C) Quantitative analysis of the data from (B), showing the density of IκB relative to that of the loading control (GAPDH). The data are presented as the mean ± S.E.M. n = 3, *p<0.05, one-way ANOVA. (D) BV2 cells were treated with rotenone for 6 hours or LPS for 1 hour. The cells were fixed and labeled with DAPI (blue) or anti-p65 antibodies (red). The scale bar represents 10 µm. (E) The cytoplasmic and nuclear fractions from the treated BV2 cells were immunoblotted with anti-p65, anti-GAPDH or anti-Max antibodies. (F) Quantitative analysis of the data from (E), showing the density of nuclear p65 relative to that of the loading control (Max). The data are presented as the mean ± S.E.M. n = 3, *p<0.05, **p<0.01, one-way ANOVA.

NF-κB is an inducible transcription factor that is involved in many inflammatory processes, including microglial activation. IκB is an inhibitor of NF-κB [24]. IκB is ubiquitinated by SCF-type E3-ligase complexes and rapidly degraded by the ubiquitin-proteasomal system (UPS) after phosphorylation by IκB kinase (IKK), resulting in activation of NF-κB [25]. To test whether rotenone could activate NF-κB in microglial cells, we first examined the effect of rotenone on IKK activation in microglia. We found that phospho-IKK (pIKK) was not detected in the untreated BV2 cells but was significantly increased in BV2 cells after LPS treatment, as well as after rotenone treatment with a dose greater than 0.5 µM (Fig. 1A). Similar to LPS, treatment with a high dose of rotenone induced the degradation of IκB in BV2 cells (Fig. 1B, C). These data suggest that rotenone directly induces IKK activation and IκB degradation in BV2 cells.

The NF-κB p65 subunit was located in the cytoplasm when BV2 cells were untreated or treated with the lower dose of rotenone. In contrast, p65 was observed in both the cytoplasm and the nucleus in the LPS-treated BV2 cells and those treated with the higher dose of rotenone (Fig. 1D), suggesting that rotenone, like LPS, can induce nuclear translocation of NF-κB from the cytoplasm to the nucleus. To further verify that rotenone induced nuclear translocation of p65, we performed a nuclear extraction experiment. We detected a significant increase in nuclear p65 in the BV2 cells after treatment with rotenone or LPS (Fig. 1E, F). Thus, our data indicate that rotenone can directly induce NF-κB activation in BV2 cells.

### Rotenone Induced the Production of Inflammatory Factors in BV2 Cells

**Figure 2:**
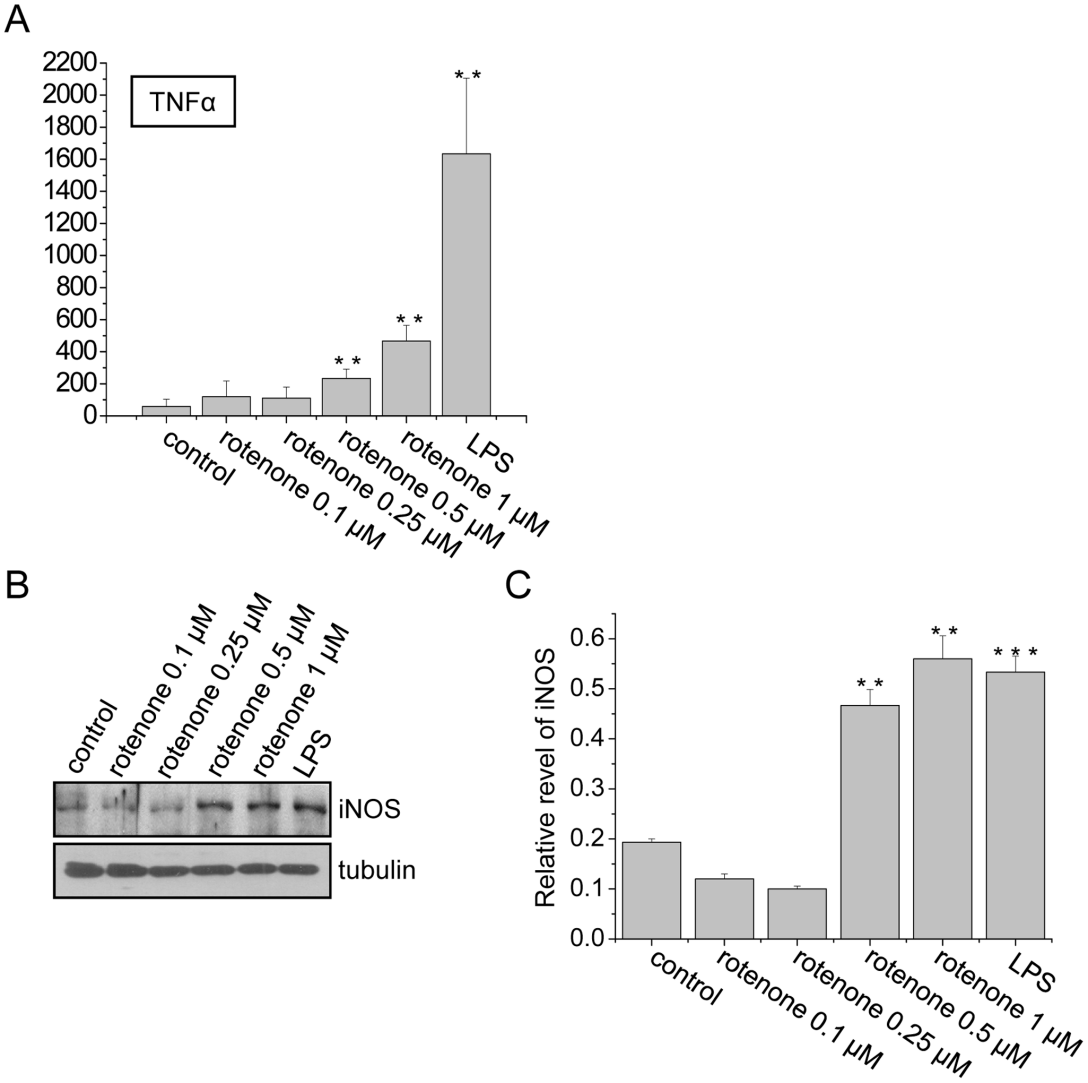
Rotenone induced inflammatory cytokine expression in BV2 cells. (A) BV2 cells were treated with different doses of rotenone or LPS for 24 hours. The TNFα levels in the cultured media were measured using ELISA assays. The data are presented as the mean ± S.E.M. n = 3, **p<0.01, one-way ANOVA. (B) Immunoblot analysis to detect iNOS expression in the BV2 cells treated with rotenone or LPS. (C) Quantitative analysis of the data from (B), showing the density of iNOS relative to that of the loading control (tubulin). The data are presented as the mean ± S.E.M. n = 3, **p<0.01, ***p<0.001, one-way ANOVA.

When p65 translocates to the nucleus from the cytoplasm, it promotes the expression of many genes, including inflammatory factors such as inducible NO synthase (iNOS) and tumor necrosis factor-α (TNF-α) [26]. This expression is a key element of inflammatory cell activation. In BV2 cells treated with a high dose of rotenone, significant production of TNF-α was detected using an ELISA assay (Fig. 2A), and iNOS levels were significantly increased 24 hours after treatment (Fig. 2B, C).

### Rotenone Induced Inflammasome Activation in BV2 Cells

The activated NF-κB signaling pathway has been suggested to induce inflammasome activation by elevating the expression of the inflammasome components. The assembled inflammasomes eventually induce caspase-1 cleavage and activation. Subsequently, activated caspase-1 cleaves pro-IL-1β to produce mature IL-1β, which is also a potent activator of the NF-κB signaling pathway, resulting in a positive feedback loop and an enhanced immune response [27]. We therefore examined whether the rotenone-activated NF-κB signaling pathway induces inflammasome assembly in BV2 cells.

**Figure 3:**
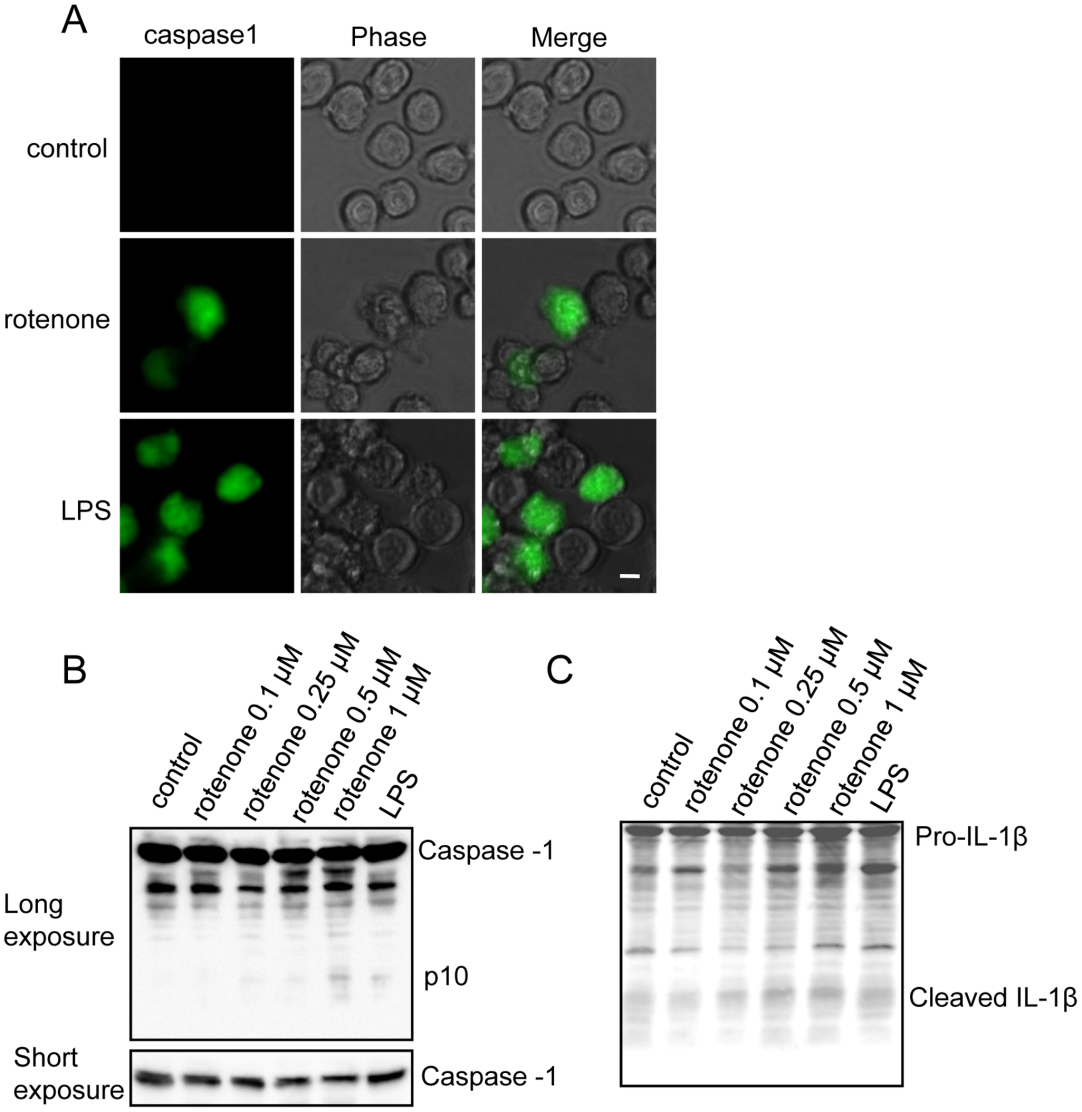
Rotenone induced inflammasome activation in BV2 cells. (A) BV2 cells were treated with 1 µM rotenone or 1 µg/mL LPS for 24 hours and then stained with green fluorescent probes for activated caspase-1 for 1 hour. The scale bar represents 10 µm. (B) BV2 cells were treated with different doses rotenone or LPS, as indicated for 24 hours. The cell lysates were immunoblotted using anti-caspase-1 antibodies. The long-exposure film showed the cleaved caspase-1 (the active p10 form) in the BV2 cells treated with the higher doses of rotenone or LPS. The total caspase-1 levels are shown in the short-exposure film. (C) BV2 cells were treated in the same manner as those in (B). The cell lysates were immunoblotted with anti-IL-1β antibodies. The activated IL-1β cleaved by caspase-1 is indicated as cleaved IL-1β.

We used a green fluorescent indicator (FAM-FLICA in vitro caspase-1 kits) that can enter each cell and irreversibly bind to activated caspase-1 to indicate inflammasome activation. We found that caspase-1 was significantly activated in the BV2 cells treated with rotenone or LPS (Fig. 3A). The activation of caspase-1 by rotenone was further confirmed using immunoblot analysis, which showed that cleaved caspase-1, in the active p10 form, was present in the BV2 cells treated with LPS or 1 µM rotenone. In contrast, activated caspase-1 was not detected in cells that had not been treated with rotenone or those treated with a low dose (Fig. 3B). Mature IL-1β, a cleaved form of pro-IL-1β, was also detected in the caspase-1-activated BV2 cells (Fig. 3C). Thus, these results suggest that rotenone activates the NF-κB signaling pathway and inflammasome in microglia.

### Rotenone Regulated the NF-κB Signaling Pathway through p38

**Figure 4:**
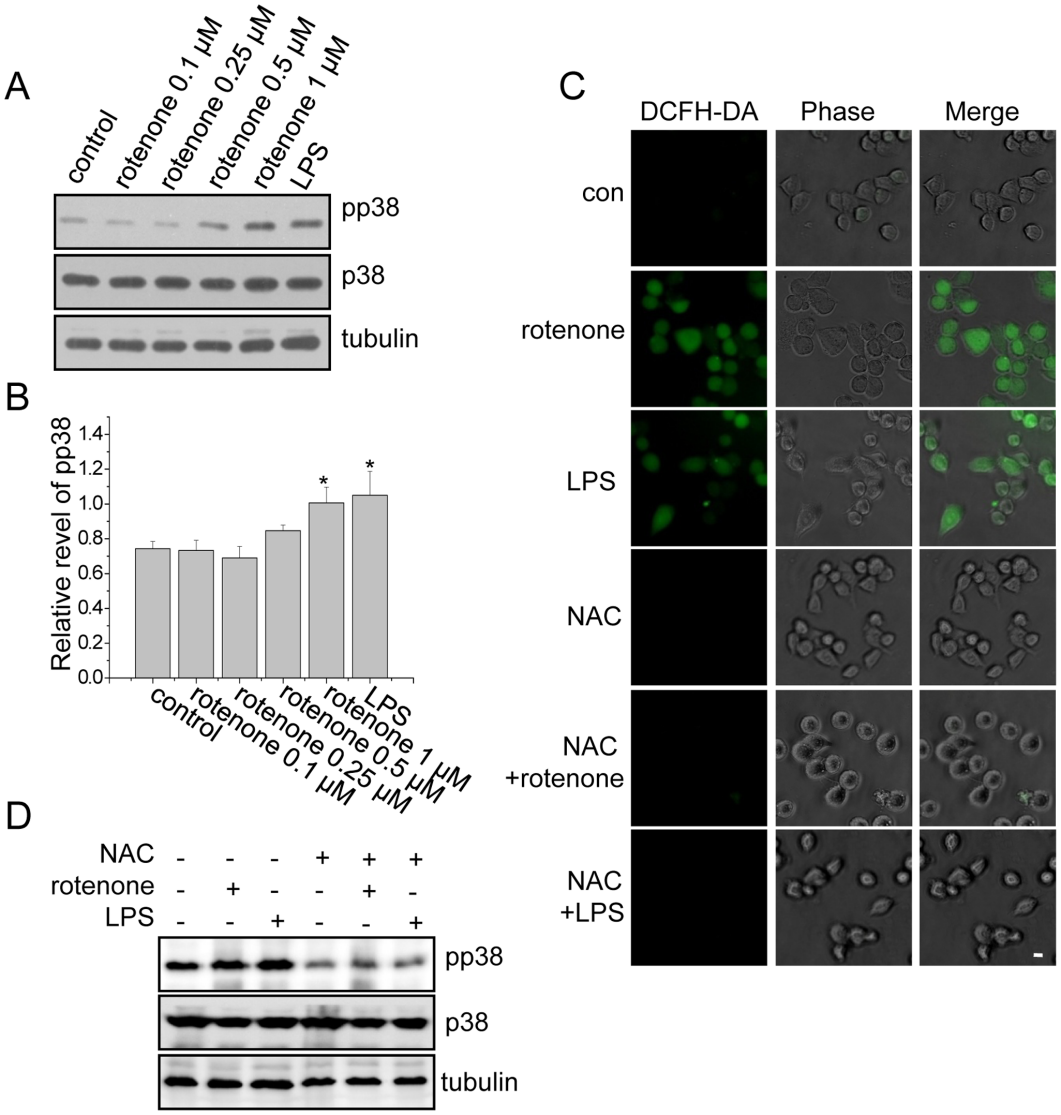
Rotenone induced microglial activation via p38 MAPK. (A) BV2 cells were treated with different doses of rotenone for 6 hours or 1 µg/mL LPS for 1 hour and lysed in lysis buffer. The lysates were immunoblotted with the indicated antibodies. (B) Quantitative analysis of the data from (A), showing the density of pp38 relative to that of the loading control (tubulin). The data are presented as the mean ± S.E.M. n = 3, *p<0.05, one-way ANOVA. (C) BV2 cells were treated with 1 µM rotenone for 6 hours or 1 µg/mL LPS for 1 hour or co-treated with 5 mM NAC and either 1 µM rotenone for 6 hours or 1 µg/mL LPS for 1 hour. The cells were stained with an ROS indicator, DCFH-DA, for 30 minutes. The scale bar represents 10 µm. (D) BV2 cells were treated with rotenone or LPS in the same manner described in (C). The cells were lysed, and the lysates were immunoblotted with the indicated antibodies.

Many studies have shown that LPS-induced NF-κB activation is dependent on p38 MAPK and that inhibiting the phosphorylation of p38 can significantly block NF-κB activation in inflammatory cells [28], [29]. We therefore tested whether the rotenone-induced NF-κB signaling pathway is dependent on p38. In rotenone-treated BV2 cells, the phosphorylation of p38 significantly increased as the dose of rotenone increased (Fig. 4A, B). In contrast, two other MAPKs, ERK and JNK, were not activated by rotenone in BV2 cells (data not shown).

Because p38 has been reported to be phosphorylated in response to ROS-induced oxidative stress [30], [31], we wondered whether rotenone-induced p38 activation was associated with ROS production. We found that both rotenone and LPS induced ROS production in BV2 cells (Fig. 4C). Furthermore, in cells treated with N-acetylcysteine (NAC), an ROS scavenger, the production of ROS and the phosphorylation of p38 that were induced by rotenone were blocked (Fig. 4C, D). Interestingly, LPS-induced p38 activation was also blocked by NAC (Fig. 4D). These results suggest that both rotenone- and LPS-induced p38 activation are dependent on ROS.

### NAC Inhibited the Rotenone-induced Activation of the NF-κB Signaling Pathway

**Figure 5:**
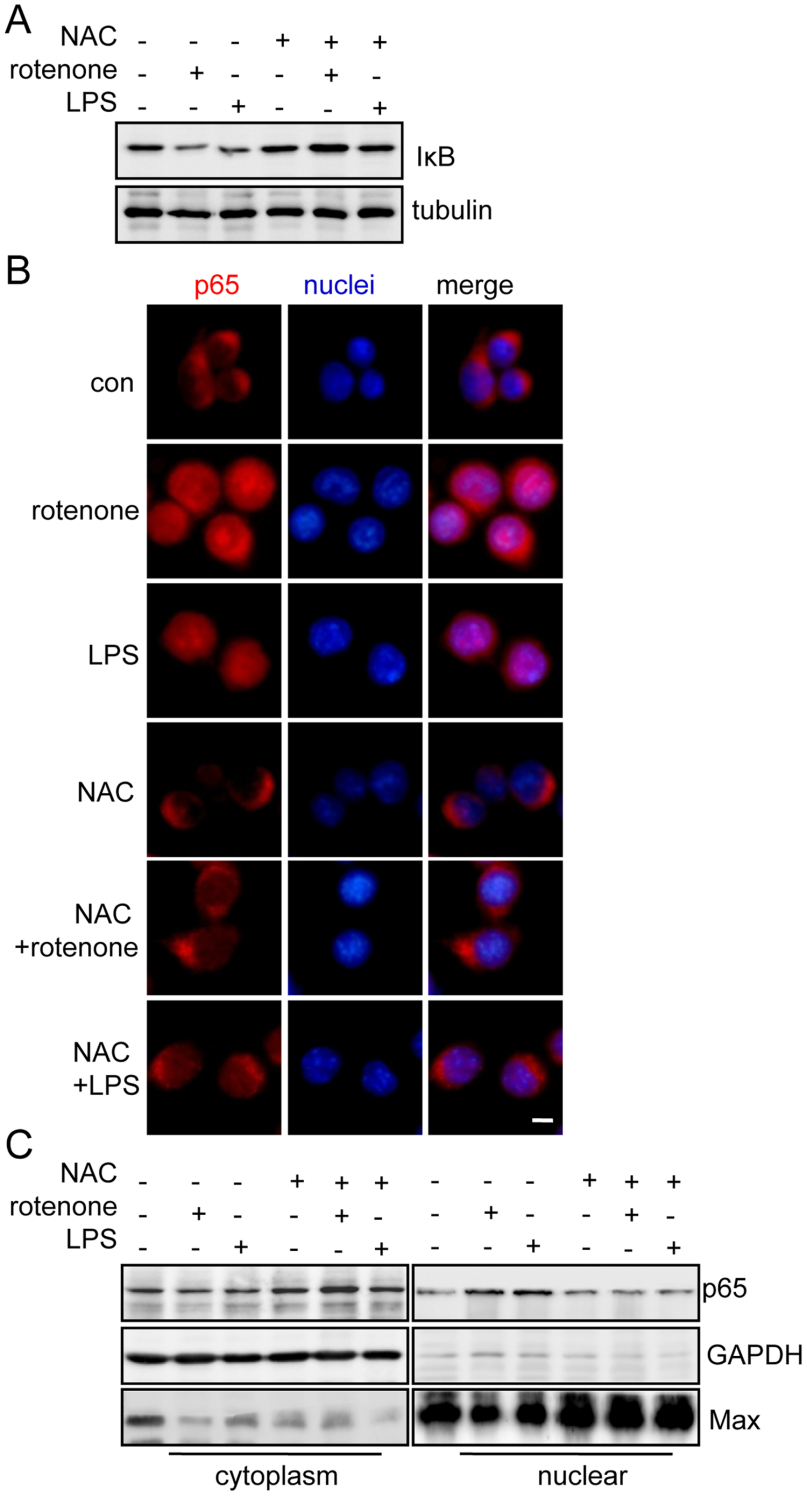
NAC blocked activation of the rotenone-induced NF-κB signaling pathway. (A) BV2 cells treated with 1 µM rotenone for 6 hours or 1 µg/mL LPS for 1 hour or co-treated with 5 mM NAC and either 1 µM rotenone for 6 hours or 1 µg/mL LPS for 1 hour. The cells were immunoblotted with anti-IκB antibodies. Tubulin served as the loading control. (B) BV2 cells treated with 1 µM rotenone for 6 hours or 1 µg/mL LPS for 1 hour or co-treated with 5 mM NAC and either 1 µM rotenone for 6 hours or 1 µg/mL LPS for 1 hour. The cells were fixed and labeled with DAPI (blue) and anti-p65 antibodies (red). The scale bar represents 10 µm. (C) The cytoplasmic and nuclear fractions from the BV2 cells that were treated as indicated in (B) were subjected to immunoblot analysis with anti-p65 antibodies. GAPDH served as the marker for the cytoplasmic fraction, and Max served as the marker for the nuclear fraction.

To further investigate whether NAC can block rotenone-induced microglial activation, we examined the NF-κB signaling pathway in BV2 cells treated with rotenone along with NAC. In the BV2 cells treated with NAC, rotenone or LPS did not induce the degradation of IκB (Fig. 5A). In addition, the p65 subunit of NF-κB was not translocated to the nucleus in the cells treated with rotenone or LPS after the administration of NAC (Fig. 5B). The nuclear fractionation experiments further confirmed the results of the immunocytochemical study, showing that p65 was mainly distributed in the cytoplasm in the BV2 cells that were treated with the combination of NAC and rotenone or LPS (Fig. 5C). Thus, these results suggest that inhibiting ROS production can block the activation of NF-κB by rotenone or LPS.

### NAC Blocked Rotenone-induced Microglial Activation

**Figure 6:**
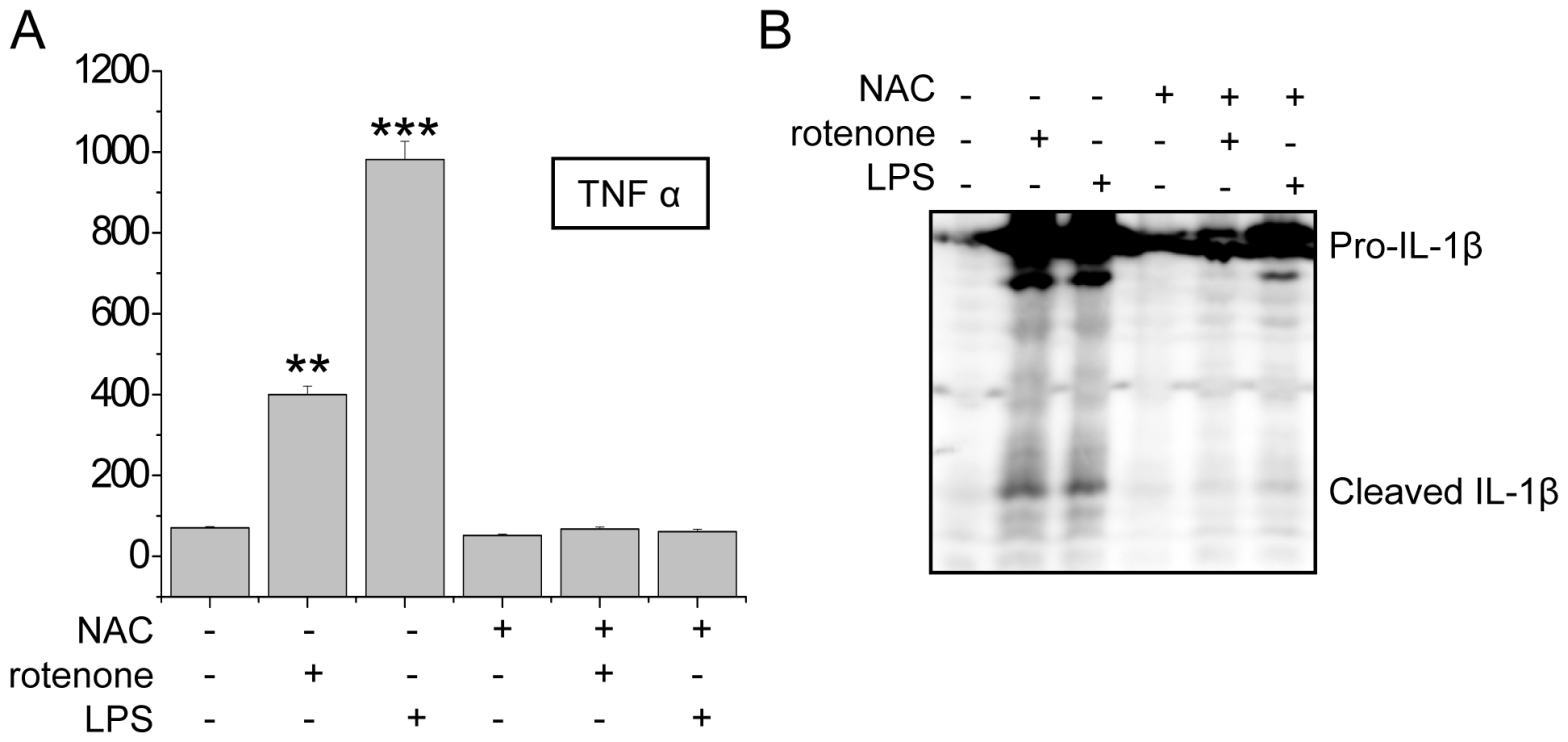
NAC blocked the rotenone-induced microglial activation. (A) BV2 cells treated with 1 µM rotenone for 24 hours or 1 µg/mL LPS for 24 hours or co-treated with 5 mM NAC and either 1 µM rotenone for 24 hours or 1 µg/mL LPS for 24 hours. The TNFα levels in the culture media of the treated BV2 cells were measured by ELISA. (B) BV2 cells were treated with as indicated in (A). Then, the cells were lysed, and the lysates were immunoblotted with the indicated antibodies. The activated IL-1β cleaved by caspase-1 is indicated as cleaved IL-1β.

Given the above observations that NAC could block NF-κB activation, we wondered whether NAC could reduce microglial activation in the cells treated with rotenone or LPS. We first measured inducible inflammatory cytokines, such as TNFα, in the culture media of BV2 cells after treatment with NAC and LPS or rotenone. We found that the production of TNFα was completely blocked by NAC in the BV2 cells treated with rotenone or LPS (Fig. 6A). Moreover, the inflammasome activation induced by rotenone or LPS, which was indicated by IL-1β cleavage, was also not detected in the NAC-treated BV2 cells (Fig. 6B). Thus, our results suggest that NAC can prevent the microglial activation induced by rotenone or LPS.

## Discussion

Several lines of evidence suggest that some molecular and cellular changes, including mitochondrial dysfunction, oxidative stress and excitotoxicity, are involved in the pathogenesis of PD. However, the underlying mechanism that induces DA neuronal degeneration is still unclear. Recently, studies have shown that neuroinflammation may contribute to the degeneration of DA neurons and that inhibiting neuroinflammation can significantly delay the progression of PD. Thus, neuroinflammation plays an important role in the pathogenesis of PD.

In the present study,we observed that rotenone, an environmental toxin that induces mitochondrial dysfunction, activated the NF-κB signaling pathway in BV2 cells. The NF-κB signaling pathway is the pivotal pathway for immune cell activation. The activation of NF-κB depends on its nuclear translocation, and activated NF-κB transactivates many proinflammatory genes. Phosphorylation of IKK, an important step for NF-κB activation, initiates the phosphorylation and degradation of IκB (inhibitor of NF-κB). Our observations indicated that rotenone induced the phosphorylation of IKK, the degradation of IκB and the nuclear translocation of the NF-κB p65 subunit. Thus, our results suggest that rotenone can directly induce NF-κB activation in microglia cells.

Activation of the NF-κB signaling pathway in immune cells induces significant production of inflammatory cytokines, such as TNF-α. We consistently found that rotenone induced a significant increase in TNF-α. Additionally, iNOS, another inflammatory cytokine, was also induced by rotenone in BV2 cells. Furthermore, we found that rotenone induced the formation of mature IL-1β and the activation of caspase-1 in BV2 cells. Pro-IL-1β was cleaved by caspase-1 to form mature IL-1β when the inflammasome was activated. Therefore, our data suggest that rotenone can activate the inflammasome in BV2 cells.

Our observations indicate that rotenone-induced NF-κB activation is strictly dependent on p38 MAPK. Rotenone induced significant ROS production and p38 activation. Interestingly, we found that LPS also induced ROS production and p38 activation. The activation of both the NF-κB signaling pathway and p38 was blocked when we used NAC to scavenge the ROS that were induced by rotenone or LPS. Moreover, the production of inflammatory factors and the activation of caspase-1 were completely inhibited in BV2 cells co-treated with NAC and rotenone or LPS. Thus, the microglial activation induced by rotenone was dependent on p38 activation, and ROS was an important mediator for rotenone- or LPS-induced NF-κB activation.

In the present study, we found that rotenone directly activated the NF-κB signaling pathway in BV2 cells. The activated NF-κB signaling pathway induced significant inflammatory cytokine production and microglial activation. The activation of the NF-κB signaling pathway was strictly dependent on p38 MAP kinase. Rotenone inhibited the complex I activity and induced significant ROS production to activate p38. Thus, our study revealed a role for environmental toxins in microglial activation. This result may help explain the presence of microglial activation in sporadic PD patients.
